# Abnormal degree centrality in delayed encephalopathy after carbon monoxide poisoning: a resting-state fMRI study

**DOI:** 10.1007/s00234-020-02369-0

**Published:** 2020-01-18

**Authors:** Kaifu Wu, Meng Liu, Laichang He, Yongming Tan

**Affiliations:** Department of Radiology, The First Affiliated Hospital of Nanchang University, Nanchang, 330006 Jiangxi China

**Keywords:** Brain, Delayed encephalopathy after carbon monoxide poisoning, Degree centrality, Resting-state, Functional magnetic resonance imaging

## Abstract

**Purpose:**

To explore neuropathologic mechanisms in functional brain regions in patients with delayed encephalopathy after carbon monoxide poisoning (DEACMP) from the perspective of the brain network nodes by resting**-**state functional magnetic resonance imaging (rs-fMRI).

**Methods:**

The fMRI and cognitive assessments were performed in 25 patients with DEACMP and 25 age-, sex- and education-matched healthy controls (HCs). Data analysis was performed via the degree centrality (DC) method. Then, the associations between the cognitive assessments and DC in the identified abnormal brain regions were assessed by using a correlation analysis.

**Results:**

Compared with the HCs, the DEACMP patients displayed significantly decreased DC values in the right superior frontal gyrus, right precentral gyrus, right angular gyrus, right marginal gyrus, right hippocampus, and left thalamus but increased DC values in the right inferior frontal gyrus, right cingulate gyrus, left superior temporal gyrus, left medial temporal gyrus, right lingual gyrus, and right posterior cerebellar lobe, pons, and midbrain (GRF correction, voxel *P* value < 0.001, cluster *P* value < 0.01). The correlation analysis in the DEACMP group revealed that there was a negative correlation between the DC values in the right hippocampus and MMSE scores, whereas a positive correlation was observed in the right cingulate gyrus.

**Conclusions:**

Patients with DEACMP exhibited abnormal degree centrality in the brain network. This finding may provide a new approach for examining the neuropathologic mechanisms underlying DEACMP.

## Introduction

Carbon monoxide (CO) poisoning is not rare and the incidence of delayed encephalopathy after carbon monoxide poisoning (DEACMP) is relatively high. Approximately 13–50% of patients with severe CO poisoning suffer from memory impairment, cognitive dysfunction, and behavioral disorders after a lucid interval (usually 2 to 60 days) of normal or apparent recovery [1–3], and the patients’ condition was evaluated by clinical cognitive tests. This complication in clinical practice is considered DEACMP [4], which is characterized by a long course, poor prognosis, and high disability rate. Partly due to the unclear pathogenesis, there is still no specific method for the treatment of this disease. Clinicians and researchers have recently suggested that early hyperbaric oxygen (HBO) therapy may be effective for the disease, as this strategy has the potential to reduce the severity of this debilitating disorder or delay or even prevent relapse [5]. Therefore, the early assessment of brain abnormalities in patients with CO poisoning is crucial for the prompt treatment of these patients and the improvement of their prognosis [6].

Some clinical obstacles exist to the assessment of brain damage in DEACMP patients. First, predicting clinical behaviors such as specific types of chronic neuropsychiatric symptoms is difficult. Second, damage to the brain is difficult to objectively assess by conventional MR imaging. Serial DWI and ^1^H-MRS measurements are useful in determining the tissue damage and long-term outcomes of delayed sequelae associated with the interval form of CO poisoning [7]. ^1^H MRS is the most useful indicator in the clinical evaluation of patients with the interval form of CO poisoning compared with EEG and SPECT [8]. A DTI study suggested that reduced connectivity between different cortical regions is a pathophysiologic mechanism for cognitive deficits in DEACMP [9]. DTI and MR spectroscopies in the subacute or chronic phase have previously revealed slight pathologic changes in progressive demyelination starting immediately after CO inhalation.

Functional MRI (fMRI) detecting the spontaneous neuronal activity in the human brain by measuring changes associated with the blood oxygenation level–dependent(BOLD) signal has become one of the commonly used methods to study brain function changes in recent years [10]. The human brain is a complex network system, and the network is a collection of nodes and edges, where nodes refer to the basic elements of the brain network and the edges are the degree of correlation between these elements [11]. Degree centrality (DC) of rs-fMRI describes the extent of centrality of the network by assessing the nodes and is the most direct analytical method for describing the impact and function of the nodes. In this study, we hypothesized that DEACMP patients had abnormal brain network nodes and compared them with the HCs’ brain function network to explore the characteristics of brain network function damage and cognitive impairment–related mechanisms in DEACMP patients.

## Methods

### Subjects

Twenty-five patients (14 males, 11 females; mean age: 38.20 ± 5.34 years) with acute CO poisoning and subsequently progressed to DEACMP were enrolled in our study from January 2016 to December 2018. Patients meeting the following diagnostic criteria were selected: (1) patients had a clear history of acute CO poisoning; (2) there was a distinct “lucid interval” of 2–60 days, with normal or near-normal performance;(3) the consciousness disorder was caused by acute CO poisoning and included neuropsychiatric symptoms, pyramidal neurologic damage, and extrapyramidal manifestations; and (4) there was no cerebral hemorrhage, cerebral infarction, or other intracranial lesions. Meanwhile, 25 age-, sex- and education-matched healthy subjects were enrolled as controls for the study. All were right-handed. The study was approved by the Ethics Committee of the First Affiliated Hospital of Nanchang University, and all participants or family members gave written informed consent before study inclusion.

### Application of standard clinical cognitive function scales

1. The Mini-Mental State Examination (MMSE) consists of 11 items (maximum score 30) covering various cognitive domains, including orientation, memory, and language. Since the MMSE is a brief screening test, the visuospatial abilities and execution are only superficially assessed. The following cutoff scores have been consensually and extensively used: (a) for patients who were illiterate, scores ≤ 17; (b) for those with 1 to 6 years of schooling, scores ≤ 20; and (c) for those with more than 6 years of schooling, scores ≤ 23; a cutoff score of < 24 was used as a value indicative of likely cognitive impairment. The lower the score was, the more severe the cognitive impairment.
2. The Montreal Cognitive Assessment (MoCA) has a high sensitivity for detecting mild cognitive impairment (MCI), and it has become increasingly prevalent and useful as an alternative tool for the traditional MMSE. The MoCA examines different cognitive abilities, such as visuospatial/executive function, naming, attention, language, abstraction, delayed recall, and orientation. It is scored out of 30 points, with higher scores reflecting better performance. The original validation study of the MoCA suggested a cutoff score of 26, with those scoring 25 or below suspected of having MCI. The authors also suggested that people being evaluated with the MoCA receive an extra point to the total score if they had ≤ 12 years of education.

### MRI acquisition procedures

Magnetic resonance imaging was performed using a 3.0-T scanner (Siemens Trio Tim, Erlangen, Germany) with an eight-channel head coil. During the scanning, the participants were instructed to stay relaxed, stay awake with their eyes closed and remain as motionless as possible. Earplugs and foam pads are used to reduce scanner noise and minimize head movement. First, high-resolution anatomic images using a 3D T1-weighted spoiled gradient recall (3D T1-SPGR) sequence were acquired with the following parameters: repetition time (TR) = 1900 ms, echo time (TE) = 2.26 ms, flip angle (FA) = 9°, field of view (FOV) = 250 × 250 mm, matrix = 256 × 256, 176 slices, slice thickness = 1 mm, voxel size = 1.0 × 1.0 × 1.0 mm^3^, and interslice gap = 0.5 mm. For the functional scans, the parameters were TR/TE = 2000/30 ms, flip angle = 90°, FOV = 200 × 200 mm, matrix = 64 × 64, voxel size = 3.0 × 3.0 × 4.0 mm^3^, slice thickness = 4.0 mm, gap = 1.2 mm, voxel size = 3.0 × 3.0 × 4.0 mm3, and 240 time points (8 min, 6 s).

### MRI preprocessing

Preprocessing of the raw data was performed with the Data Processing Assistant for Resting-State fMRI (DPARSF), V2.1 software package (http://www.restfmri.net), which was based on the MATLAB7.8 (Math-works, Natick, MA, USA) platform. Preprocessing included the following steps: removal of the first 10 time points to avoid transient signal instability; slice timing correction, head motion correction (the data were discarded if head motion exceeded 3 mm of translation or 3° of rotation in any direction); normalization into the Montreal Neurological Institute (MNI) space (resampling voxel size = 3 mm × 3 mm × 3 mm), removal of linear trends, filtering (0.01~0.08 Hz) and nuisance covariate regression (including white matter, CSF, and the head movement parameters). After these preprocessing steps, fMRI data were used for DC calculations: Pearson’s correlation analysis of time series was executed between each voxel and every other voxel in the entire brain. Correlation coefficients with *r* > 0.25 were summed for each voxel, then the binary DC was obtained for each gray matter voxel [12]. Subsequently, spatial smoothing was performed with a 6-mm FWHM Gaussian kernel [13]. The weighted DC of each voxel was further divided by the global mean DC for standardization.

### Statistics

To detect differences in the demographic and clinical data, we compared the age, sex, years of education, MMSE score, MoCA score between the two groups by conducting one-way analysis of variance (ANOVA), and the *χ*^2^ test using SPSS software version 24.0. Then, two-sample *t* tests were performed to probe the group differences in the DC maps across the groups with age, sex, and years of education as covariates. The resultant T-maps were corrected using the Gaussian random field (GRF) method (voxel *P* value < 0.001, cluster *P* value < 0.01). Finally, to determine whether abnormal DC was associated with the clinical assessment (measured by MMSE and MoCA scores) of the DEACMP patients, we conducted a Pearson correlation analysis. A threshold of *P* = 0.05 was used to determine the significance level.

## Results

### Demographic statistics between groups

The distribution of MMSE and MoCA scores in the DEACMP group was lower than those in the HC group, especially in visual space/execution and delayed memory (*P* < 0.01), indicating that impairment in visual space/execution, as well as delayed memory, was most prominent. These manifestations were often the early symptoms of delayed encephalopathy caused by CO poisoning. And the data from the scale reflected a degree of cognitive dysfunction in the patients with DEACMP. There was no significant difference in age, sex, or years of education between the groups (all *P* > 0.05) (Table 1).

**Table 1:** Demographic and clinical characteristics of DEACMP patients and HCs

	DEACMP (*n* = 25)	HCs (*n* = 25)	*P* value
Age (years)	38.2 ± 5.34	38.9 ± 2.75	0.56
Sex (males/females)	14/11	13/12	0.77
Education (years)	12.5 ± 3.26	13.9 ± 4.15	0.191
MMSE	20.68 ± 2.53	27.80 ± 1.41	< 0.01
MoCA	18.21 ± 3.21	26.02 ± 3.55	< 0.01
Visual space/execution	2.38 ± 1.05	3.55 ± 0.52	< 0.01
Delayed memory	3.02 ± 0.54	3.59 ± 0.28	< 0.01

### Comparison of DC values between the DEACMP group and HC group

**Fig. 1:**
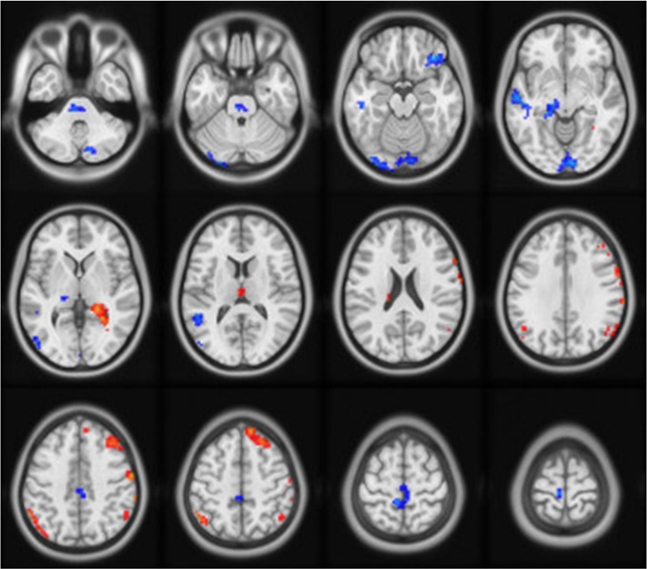
The group differences in DC between DEACMP patients and healthy controls. Red and blue denote higher and lower DC in patients than in healthy controls

Compared with HC group, the DEACMP group had significantly decreased DC in the right superior frontal gyrus, right precentral gyrus, right angular gyrus, right marginal gyrus, right hippocampus, and left thalamus. The patients with DEACMP also exhibited some areas with increased DC compared with healthy controls. These regions included the right inferior frontal gyrus, right cingulate gyrus, left superior temporal gyrus, left medial temporal gyrus, right lingual gyrus, and right posterior cerebellar lobe, pons, and midbrain (Fig. 1, Table 2).

**Table 2:** Distribution of brain regions with significant differences in DC values between the DEACMP group and HC group

Brain region	Brodmann area	Peak MNI coordinate			Voxels	Peak *T* value
		*X*	*Y*	*Z*		
DEACMP < HCs						
Right superior frontal gyrus	8	9	48	48	239	5.77
Right precentral gyrus	6	63	− 9	39	102	7.64
Right angular gyrus	39	57	− 60	27	31	4.79
Right marginal gyrus	40	63	− 39	48	42	5.52
Right hippocampus	-	18	− 33	6	133	6.37
Left thalamus	-	− 3	− 18	15	36	4.72
DEACMP > HCs						
Right inferior frontal gyrus	47	36	33	− 18	67	− 5.65
Right cingulate gyrus	31	3	− 30	45	173	− 5.08
Left superior temporal gyrus	22	− 54	− 54	9	208	− 6.22
Left medial temporal gyrus	19	− 51	− 75	9	44	− 4.70
Right lingual gyrus	18	9	− 93	− 9	241	− 5.46
Right posterior cerebellar lobe	-	9	− 75	− 33	51	− 4.92
Pons	-	− 6	− 24	− 33	87	− 4.99
Midbrain	-	− 15	− 27	− 3	99	− 6.73

Note: MNI coordinates are from standard human brains from the Montreal Neurological Institute. This table shows the peak voxel coordinates; the positive peak intensity represents an increase in the DC value, and a negative value represents a decrease in the DC value. GRF correction at the cluster level with voxel *P* of 0.001 and cluster *P* of 0.01

### DEACMP group DC value change and MMSE and MoCA scale correlation analysis

**Fig. 2:**
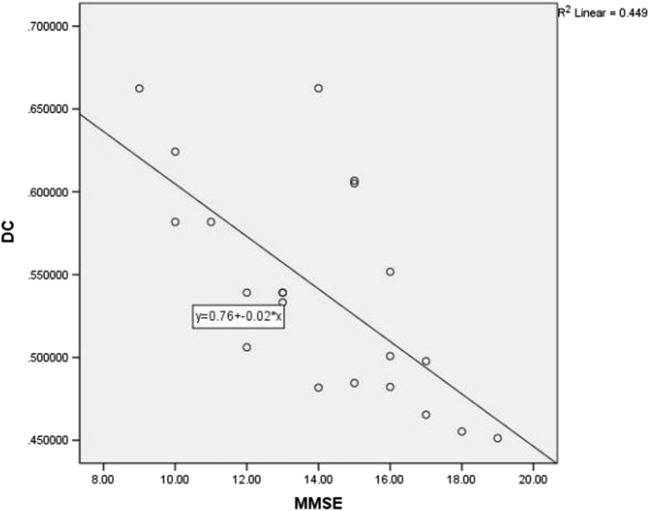
Correlation analysis between the DC values of the right hippocampus and MMSE scores in the patients with DEACMP

**Fig. 3:**
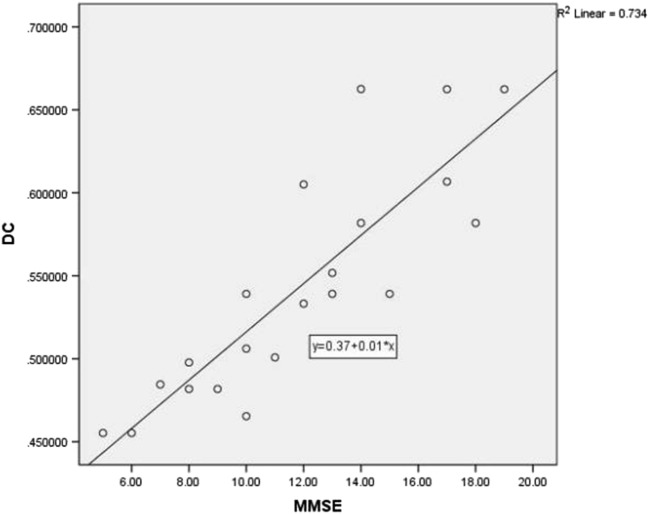
Correlation analysis between the DC values of the right cingulate gyrus and MMSE scores in the patients with DEACM

Compared with the DC values in the HCs, the DC values of the different brain regions in the DEACMP group were correlated with the cognitive assessment of the patients. Through analysis, the DC values of the right hippocampus showed a negative correlation with the MMSE scores (*r* = − 0.670, *P* < 0.01) (Fig. 2, Table 3), whereas there was a positive correlation between DC values of the right cingulate gyrus and MMSE scores (*r* = 0.857, *P* < 0.01) (Fig. 3, Table 3). However, we did not find any significant correlation between cognitive scores and abnormal DC in the remaining brain regions.

**Table 3:** Correlation analysis between the right hippocampus and cingulate gyrus DC values and MMSE scores in the patients with DEACMP

Brain region	MMSE score	
	*r*	*P*
Right hippocampus	− 0.670	< 0.01
Right cingulate gyrus	0.857	< 0.01

## Discussion

Degree centrality, a directly quantifiable index to measure the importance of brain network nodes and reflects the functional network “hub” properties, is the most reliable centrality metric in large-scale networks [14–16]. Conceptualizing the brain as a network is potentially helpful for researchers to investigate the integrated neuronal network abnormalities in the early stages of some neuropsychiatric disorders [11]. DC represents the role and status of voxels in the brain network, and changes in DC indicate an anomaly between the functional synchronization of a particular node and other nodes. For example, increased DC values suggest an increased number of direct connections and reflect the increased centrality or importance of a specific voxel in the network [17]. DC was initially proposed to map the degree of intrinsic functional connectivity across the brain to reveal a stable property of cerebral network architecture at the voxel level [15]. Previous studies using topology analysis of complex networks have shown that brain region nodes with the most connections (edges) to other regions are the ones most often damaged by disease [18]. This may be related to the higher blood flow, glucose metabolic rate, and longer connection distance in the efficient central nodes of the brain than in the noncentral nodes [19]. Focal brain damage that affects nodes involved in intermodule connectivity leads to reconfiguration of important nodes in the entire network [20], which may be associated with clinically significant cognitive impairment. Some disease processes may start in peripheral nodes and only develop symptoms after they have spread to the central node of the topology [21].

The clinical characteristic of DEACMP is the recurrence of neuropsychiatric symptoms, and its pathogenesis is complex. Studies have shown that CO poisoning will lead to increased release of glutamic acid [22] and catecholamines [23], leading to demyelination of the white matter. Some researchers have reported that white matter damage can cause interruption in remote communication between large brain regions and disrupt interactions between the nodes of the brain’s intrinsic neural network, which are the basis for cognitive dysfunction [24]. At the same time, this damage increases Bcl-2 expression, stimulates nicotinic acetylcholine receptors, activates the phosphatidylinosital-3-kinase (PI-3 K)/Akt pathway, and inhibits glycogen synthesis of kinase-3 causing neuronal apoptosis in the hippocampus and cortex [25]; through immune inflammatory responses, it dilates blood vessels, which further leads to the destruction of the blood-brain barrier, and there is damage from oxidative metabolism due to free radicals produced by hypoxia that leads to brain cell edema. The gray matter damage in the cerebral cortex may be closely related to the occurrence of clinical symptoms of DEACMP.

In detail, the results showed lower DC in the right superior frontal gyrus, right precentral gyrus, right angular gyrus, right marginal gyrus, right hippocampus, and left thalamus. Here, injury in network nodes was interpreted as a reduction in the influence of local network communication on other parts of the network, suggesting a breakdown in the nodes’ functional interactions in a network, which can be a sign of decompensation or imbalance. DC was increased in the right inferior frontal gyrus, the right cingulate gyrus, the left superior temporal gyrus, the left medial temporal gyrus, the lingual gyrus of the right occipital lobe, the right posterior cerebellar lobe, and the left side of the pons and midbrain, which meant increased importance of these regions in the brain of patients with DEACMP [16]. One explanation for the higher degree centrality values could be that the hyperactivation in these areas may be interpreted as an enhanced neural effort to offset structural damage to the brain [26]; the gradual decompensation leads to a reconfiguration of important nodes in the whole network, causing the symptoms to further aggravate, which is a process of continuous progress. Previous studies have reported that limbic systems, such as the hippocampus and cingulate gyrus, are essential for the coordination of information flow throughout the brain network, and lesions in these regions will seriously affect the stability of brain system function [27]. It is known that the angular gyrus and the supramarginal gyrus belong to Wernicke’s area, which is the location of the auditory and visual language centers; the decrease in DC in these brain regions may indicate that patients also have language and visual dysfunction. Singhal et al. [28] reported a case of acute CO poisoning in patients exposed to CO gas for 3 days. The diffusion MRI showed decreased ADC values in the hippocampus, temporal lobe, and occipital cortex, which may represent early cytotoxic edema secondary to cell energy failure. However, our findings show that functional connectivity changes simultaneously appeared in these regions. Based on the functional network integrity perspective, it could be postulated that the function of cognitive control–related regions was weakening [17]. Therefore, we hypothesized that deficits in cognitive control regulatory processes might occur in DEACMP patients during the resting state. Meanwhile, this study found that the DC values of the right hippocampus and cingulate gyrus in patients with DEACMP were correlated with MMSE scores, further supporting that the above brain regions are involved in the pathophysiology of the disease. Both the hippocampus and cingulate gyrus belong to the limbic system and are interlinked through the Papez loop, which is involved in mediating emotional behavior and information exchange. Therefore, if the cingulate gyrus or the structure related to its function is damaged, it may directly or indirectly cause cognitive-related abnormalities, and preexisting synaptic connections between neurons or new neural circuits will compensate, synergistically involving the hippocampus in the remodeling of the central function of the disease. However, the study showed that, except for the above-mentioned brain regions, other abnormal brain regions in the DEACMP group were not significantly associated with the assessment of cognitive function. This may be related to the small sample size and different disease courses across patients, or perhaps these brain regions were involved in the pathological process of DEACMP-related cognitive impairment but do not fully reflect the severity of cognitive impairment. Besides, there was no significant correlation between DC values in other brain regions and the MOCA score, which may be mainly due to the different emphases of the two scales. The MMSE focuses on orientation and language function and has a low false-negative rate when assessing moderate-to-severe cognitive impairment but is vulnerable to language and education levels. In contrast, the MoCA has wider coverage and a more comprehensive assessment, especially in terms of space/execution, but its role in the diagnosis and efficacy assessment of dementia is limited. If the relevant operation can be further optimized and applied to routine screening after CO poisoning, the occurrence of DEACMP can be predicted early through changes in the DC value of brain regions, and timely clinical interventions may prevent the further progress of the disease.

The present study has some limitations. First, this study was a cross-sectional design, which cannot provide detailed information about the abnormal functional connections. Thus, future longitudinal studies should include patients with CO poisoning in different periods, as well as individuals at risk for DEACMP, to more fully reveal the pathophysiological progression of the disease. Second, the sample size was relatively small and may have limited statistical power. Therefore, more subjects are needed in future studies. Third, the white matter integrity damage caused by CO cannot be reflected by changes in DC values, so additional studies with other magnetic resonance sequences and calculation methods are needed. The mechanisms responsible for these influences and their potential implications deserve further exploration.

In conclusion, our findings show that DC values are altered in several brain regions of DEACMP patients, indicating that brain networks could be damaged and reorganized after CO poisoning, which may serve as early markers of DEACMP in patients. This resting-state fMRI study suggests a novel approach for understanding the dysfunction and pathophysiology of DEACMP.
